# Multi-level meta-workflows: new concept for regularly occurring tasks in quantum chemistry

**DOI:** 10.1186/s13321-016-0169-8

**Published:** 2016-10-20

**Authors:** Junaid Arshad, Alexander Hoffmann, Sandra Gesing, Richard Grunzke, Jens Krüger, Tamas Kiss, Sonja Herres-Pawlis, Gabor Terstyanszky

**Affiliations:** 1 Centre for Parallel Computing, School of Computer Science, University of Westminster, 115 New Cavendish Street, London, W1W 6UW UK; 2 Institut für Anorganische Chemie, Lehrstuhl für Bioanorganische Chemie, RWTH Aachen University, Landoltweg 1, 52074 Aachen, Germany; 3 University of Notre Dame, 123 Information Technology Center, Notre Dame, IN 46556 USA; 4 Center for Information Services and High Performance Computing, Technische Universität Dresden, Zellescher Weg 12-14, 01062 Dresden, Germany; 5 Applied Bioinformatics Tübingen, University of Tübingen, Sand 14, 72076 Tübingen, Germany

**Keywords:** Scientific workflows, Workflow repository, Quantum chemistry, Meta-workflow, Atomic workflow

## Abstract

**Background:**

In Quantum Chemistry, many tasks are reoccurring frequently, e.g. geometry optimizations, benchmarking series etc. Here, workflows can help to reduce the time of manual job definition and output extraction. These workflows are executed on computing infrastructures and may require large computing and data resources. Scientific workflows hide these infrastructures and the resources needed to run them. It requires significant efforts and specific expertise to design, implement and test these workflows.

**Significance:**

Many of these workflows are complex and monolithic entities that can be used for particular scientific experiments. Hence, their modification is not straightforward and it makes almost impossible to share them. To address these issues we propose developing atomic workflows and embedding them in meta-workflows. Atomic workflows deliver a well-defined research domain specific function. Publishing workflows in repositories enables workflow sharing inside and/or among scientific communities. We formally specify atomic and meta-workflows in order to define data structures to be used in repositories for uploading and sharing them. Additionally, we present a formal description focused at orchestration of atomic workflows into meta-workflows.

**Conclusions:**

We investigated the operations that represent basic functionalities in Quantum Chemistry, developed the relevant atomic workflows and combined them into meta-workflows. Having these workflows we defined the structure of the Quantum Chemistry workflow library and uploaded these workflows in the SHIWA Workflow Repository.

**Graphical Abstract:**
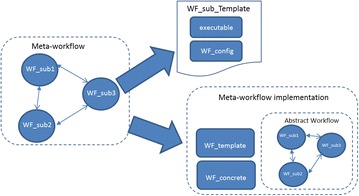
Meta-workflows and embedded workflows in the template representation

**Electronic supplementary material:**

The online version of this article (doi:10.1186/s13321-016-0169-8) contains supplementary material, which is available to authorized users.

## Background

Scientific workflows represent complex computational experiments conducted by scientists focused at identifying and addressing scientific problems across diverse subject domains such as Quantum Chemistry simulations [1], Astrophysics [2], Heliophysics [3] and Neuroimaging data analysis [4]. Such experiments usually involve analysis of large volumes of data and typically they are executed in Distributed Computing Infrastructures (DCIs), such as clouds, clusters, supercomputers, etc. as demonstrated by [5, 6]. Scientific workflows represent an abstraction that hides the complexity of the involved computing and data infrastructures. They are often composed of control and data flow statements and rules which perform the analysis required to achieve the intended experiment. A typical scientific workflow is composed of one or more distinct tasks (often termed as *jobs).* Each of these jobs performs a specific function and contributes to the overall goal of the workflow e.g., a single point energy, frequency calculation, etc. An interesting and emerging trend in workflow development is to orchestrate workflows from one or more sub-workflows, i.e. individual jobs may be workflows designed to achieve a specific function. Such composite workflows are termed as *meta*-*workflows* [7–9] and are envisaged to use existing workflows as components of the meta-workflow for improving their development and enabling their reusability. With respect to reusing multiple workflows to achieve a more complex task, terms such as *nested workflows* [10] and *embedded workflows* [11] have also been used. However, these refer to sub workflows that are collated to orchestrate meta-workflows. The development and use of meta-workflows is facilitated by repositories such as [12–14] which aim to store and share scientific workflows. The existence and flexibility of such repositories enables workflow sharing to wider scientific communities thereby facilitating development of meta-workflows to achieve modelling complex scientific problems via workflows. Meta-workflows engage complex orchestration of applications, which may span across multiple domains. For such complex workflows the workflow nodes represent a combination of jobs and sub-workflows, which can host multiple tasks within them. However, there remain challenges in achieving widespread workflow sharing as different workflow repositories may choose different approaches to describe workflows leading to problems in sharing workflows across repositories.

Computational Chemistry covers a broad range of scientific challenges and consequently a multitude of methods and algorithms have been developed over the past decades. They can be subdivided into several sub-domains including Quantum Chemistry (QC), Molecular Dynamics (MD) and Molecular Docking. Within each sub-domain simulation protocols have emerged which can be considered to be good practice within the field. These sub-domains strongly differ in theoretical approaches, simulation codes and workflows. For example in docking and Molecular Dynamics, workflows have some longer tradition [15–17] than in other sub-domains. Within the MoSGrid Science Gateway [18], which has adopted the WS-PGRADE workflow system [19], workflows have been used extensively especially in the Docking and Molecular Dynamics domain contributing to facilitated job submission and output analysis. The concept of complex meta-workflows has been recently introduced into the Quantum Chemistry sub-domain. These workflows consist of workflows with a basic set of operations that can be re-used in different complex workflows [1].

Several production workflow systems have been developed in the last decade, which serve diverse user communities, follow different workflow concepts, support different workflow languages and are based on different workflow technologies. Examples are Dispel [20], Galaxy [21], Kepler [22], KNIME [23], Pegasus [24], Swift [25], Taverna [26] and WS-PGRADE [27]. While all enable scientific workflow management, Galaxy, Kepler, KNIME, Taverna, and WS-PGRADE are widely used in the Computational Chemistry community.

WS-PGRADE is a flexible web-based user interface of the gUSE scientific gateway framework. It is built on the Liferay portal framework [28], which allows easily extending WS-PGRADE’s user interface for domain-specific features via so-called portlets and needs only installation on the server side while users can access all features via a web browser. WS-PGRADE offers workflow management features including editing, configuring, submitting and monitoring workflows plus a repository for storing workflows. This repository enables users to share workflows and to import or export workflows from and to other WS-PGRADE portals. Besides the workflow repository in WS-PGRADE, other related repositories have been implemented with complimentary features. The SCI-BUS Portlet Repository [29] has been developed to share portlets and the available user interface features provided by such extensions. The SHIWA Workflow Repository [12] follows a workflow-driven approach and allows sharing workflows between major workflow platforms such as Galaxy, Kepler, Pegasus, Taverna, WS-PGRADE, etc. There are several science gateways based on the gUSE framework in the Computational Chemistry community for example the MoSGrid portal [30], the AutoDock Portal [31] as well as the AMC Docking Gateway [32]. As above mentioned, MoSGrid supports the following three domains: Docking, Molecular Dynamics (MD) and Quantum Chemistry (QC), while the AutoDock Portal and the AMC Docking Gateway are concerned with leveraging AutoDock [33] or AutoDock Vina [34], respectively, for the Docking community. The latter portals apply pre-configured workflows similar to MoSGrid, whereas MoSGrid additionally applies the meta-workflow concept.

The KNIME workbench supports also the meta-workflow concept and enables users to easily orchestrate Computational Chemistry workflows via basic workflows in its repository. While its user interface is very intuitive, it needs installation on the user side. The KNIME Web Portal, which relieves users from the local installation, also gives access to the repository but does not possess all the features of the workbench such as reporting tools directly.

Taverna follows a similar approach compared to the KNIME workbench and requires local installations on the user’s computers. Taverna workflows can be shared in a web-based environment via the social platform myExperiment [14]. The meta-workflow concept is not supported directly in Taverna but via the web-based solution Tavaxy [7]. This workflow system has been especially created to implement the meta-workflow concept via featuring to connect Taverna and Galaxy workflows with each other and submit them. Galaxy workflows can be additionally edited in Tavaxy, while Taverna workflows can be simply re-used but not changed. Galaxy as widely used portal in the biomedical community offers also a web-based repository for sharing workflows but lacks the support of the meta-workflow concept. Further workflow solutions tailored to the Computational Chemistry community include Kepler, PyADF [35] and JACOB [36]. They are highly flexible and apply cutting edge technologies such as RESTful APIs. While the meta-workflow concept is not directly supported out of the box, they can be extended to support it. Such solutions necessitate programming skills on the users’ side though.

Our focus here is to investigate the challenges encountered in developing and using complex meta-workflows. In particular, we make the following major contributions:

- Formal definitions for atomic workflows have been formulated to facilitate their understanding and reuse by addressing challenges in workflow sharing
- A template-based approach to create complex meta-workflows has been presented along with its formal representation
- Use cases from quantum chemistry workflows have been included which represent successful demonstrations of the concepts and technologies presented herein.

## Methods

### Sharing scientific workflows

In [37] a formal description of scientific workflows was presented to enable sharing workflows inside and among research communities. This formal description defines the data and meta-data structure of scientific workflows required to manage workflows, including their uploading, editing, searching and downloading, in workflow repositories. The formal description also provides extra supports for sharing workflows of different workflow systems and their combination into meta-workflows and executing them on different DCIs.

### Atomic and compound workflows and their formal description

Scientific workflows are generally defined by four entities: abstract workflow, concrete workflow, workflow configuration and workflow engine. The abstract workflow specifies the functionality of the workflow. It defines the workflow structure as a workflow graph including its inputs and outputs, and its edges and nodes where nodes correspond to computational tasks and edges represent the control and/or data flow among nodes. It does not contain any executables, default input files and parameters needed to run the workflow. Abstract workflows may have multiple implementations defined by concrete workflows. The concrete workflow defines a workflow instance for a particular workflow engine. It delivers the functionality defined by the abstract workflow. It contains either data or references (via e.g., URLs) required to run the workflow on the associated workflow engine. Each concrete workflow has its own workflow configuration that contains parameters, references and files of the concrete workflow. Finally, the workflow engine identifies the workflow engine that executes the concrete workflow. Therefore, as described in [37], a scientific workflow can be formally defined as

1 $$ {\text{WF}} = :\left\{ {{\text{WF}}_{\text{abs}} ,{\text{WF}}_{\text{cnr}} ,{\text{ WF}}_{\text{cnf}} ,{\text{ WF}}_{\text{eng}} } \right\} $$

where WF_abs_—abstract workflow, WF_cnr_—concrete workflow, WF_cnf_—workflow configuration, WF_eng_—workflow engine.

Workflows may orchestrate complex scientific experiments with a large number of workflow nodes. These workflows are monolithic entities supporting one particular experiment. If there is need to change a scientific experiment, workflow developers have to re-design and test the whole workflow again. It may require significant efforts in both resources and time. Analysing these workflows it can be concluded that they may contain a job or a set of jobs delivering a specific functionality that can be re-used in further scientific experiments. To support workflow sharing and re-usability we introduce the concept of atomic workflows to implement modularity within scientific workflows [13, 37]. An atomic workflow is a special type of workflows, which is aimed to achieve a very specific objective delivering a specific function with a specific set of inputs and outputs. They contain only jobs i.e. they do not incorporate any further workflows. They represent a job or a set of jobs that can be re-used as part of more complex workflows. An example of such workflows can be a simple geometry optimization workflow in Computational Chemistry which can be re-used as part of a number of other possible workflows such as frequency calculation, time-dependent DFT, population analyses, etc. as demonstrated in [38]. Since atomic workflows deliver a well-defined functionality we manage them at the abstract workflow level. To manage atomic workflows both the formal definition of abstract workflows and their data structure must be extended.

In [39], we used *jobs* as a structure to represent the set of functions envisaged to be performed by a workflow. In abstract workflows *jobs* represent a set of functionalities while in concrete workflows they are the binaries that deliver these functionalities. Therefore, *f*_*h*_ *(I*_*h*_ *, O*_*h*_ *)* ∈ *jobs; h* = *1,…, k* where *f*_*h*_ represents the set of functions to be performed by a workflow. As an atomic workflow is envisaged to be focused on accomplishing a single specific function, this can be represented as *f*_*h*_ *(I*_*h*_ *, O*_*h*_ *)* ∈ *jobs; h* = *1*. As described above, this function is assumed to be generic enough to be re-used as part of more complex workflows. Now, the function *f*_*h*_ is expected to operate with a generic set of input *I,* which is envisaged to vary across application domains. Furthermore, *f*_*h*_ is expected to produce a specific set of outputs *O,* which can be consumed by other atomic workflows and/or jobs within a complex workflow. Within the context of the terminology used by workflow systems, the function *f*_*h*_ represents a ‘job’, which is envisaged to execute specific function as part of the overall workflow. We use the formalism presented in [37], to further elaborate the inputs and outputs of the atomic workflows:

atomic workflow:

2 $$ {\text{WF}}_{\text{AT}} = : \, \left\{ {{\text{job}}\left( {{\text{f}}_{\text{h}} } \right)} \right\} $$

where h = 1;

job(f_h_) ∈ *jobs*

inputs:

3 $$ I = \, \left\{ {{\text{I}}_{1} ,{\text{ I}}_{2} ,{\text{ I}}_{3} , \ldots ,{\text{I}}_{u} } \right\} $$

where I_i_—input for the workflow; i = 1,…, u

I_i_ = {input_id, input_description, input_data_type}

outputs:

4 $$ O \, = \left\{ {{\text{O}}_{1} ,{\text{ O}}_{2} ,{\text{ O}}_{3} , \ldots,{\text{ O}}_{v} } \right\} $$

where O_j_—output for the workflow; j = 1,…, v, O_j_ = {output_id, output_description, output_data_type}

Having the formal description of the atomic workflows and their functionality at the abstract workflow level we extended the data and meta-data structure of abstract workflows. (See in Table 1) These data structures enable publishing atomic workflows in workflow repositories, for example in the SHIWA Workflow Repository [12]. As a result, atomic workflows can be searched and found in these repositories enabling workflow developers to embed atomic workflows with the required functionality in meta-workflows.

**Table 1:** Data structure of the atomic workflow’s functionality in the repository

Element	Sub-element	Sub-sub-element	Data type
Functionality			Ontology vocabulary
Input(s)	input 1	input_id	Integer
		input_description	Plain text
		input data type	Data type
	input 2	input_id	Integer
		input_description	plain text
		input data type	Data type
	input…		
	input u	input_id	Integer
		input_description	Plain text
		input data type	Data type
Output(s)	output 1	output_id	Integer
		output_description	Plain text
		output data type	Data type
	output 2	output_id	Integer
		output_description	Plain text
		output data type	Data type
	output *…*		
	output v	output_id	Integer
		output_description	Plain text
		output data type	Data type

### Workflow libraries and atomic workflows

Scientific workflows represent valuable knowledge incorporating verified methods to perform specific experiments. Within this context, sharing workflows to establish and improve collaborations facilitates advancement of scientific knowledge. Workflow repositories and libraries have a profound role in achieving this objective by providing the enabling environment. To support workflow sharing we recommend creating a workflow library of atomic workflows for a specific research area, a domain, and it may have multiple sub-domains. Defining a workflow library follows a top-down approach, i.e. first the domain is identified, next, the sub-domains are defined and finally, the functionality of atomic workflows specified.

5 $$ {\text{SUB}}\_{\text{DOMAIN}}_{k} \in {\text{DOMAIN}} $$

where k = 1,…, w

6 $$ {\text{WF}}_{{{\text{AT}}({\text{l}})}} \in {\text{SUB}}\_{\text{DOMAIN}}_{\text{k}} $$

where l = 1,…, y

Being familiar with a particular research domain, researchers can identify the relevant sub-domains and define the functionality of workflow library of each sub-domain.

### Meta-workflows and their formal description

Complex workflows, also called meta-workflows, may contain jobs and workflows. We call workflows included in meta-workflows embedded workflows. We consider atomic workflows as a sub-set of embedded workflows.

7 $$ {\text{M}{-}\text{WF}} = :\left\{ {{\text{J}}_{1} \ldots .{\text{J}}_{{{\text{m}} + {\text{n}}}} } \right\} = \left\{ {{\text{J}}_{{{\text{WF}}1}} \ldots {\text{J}}_{\text{WFm}} ,\;{\text{WF}}_{{{\text{EM}}1}} \ldots {\text{WF}}_{\text{EMn}} ,} \right\} $$

where J_WFm_—workflow job m = 1,…, z, WF_EMn_—embedded workflow n = 1, *…*, y, WF_AT_ ∈ WF_EM_

**Fig. 1:**
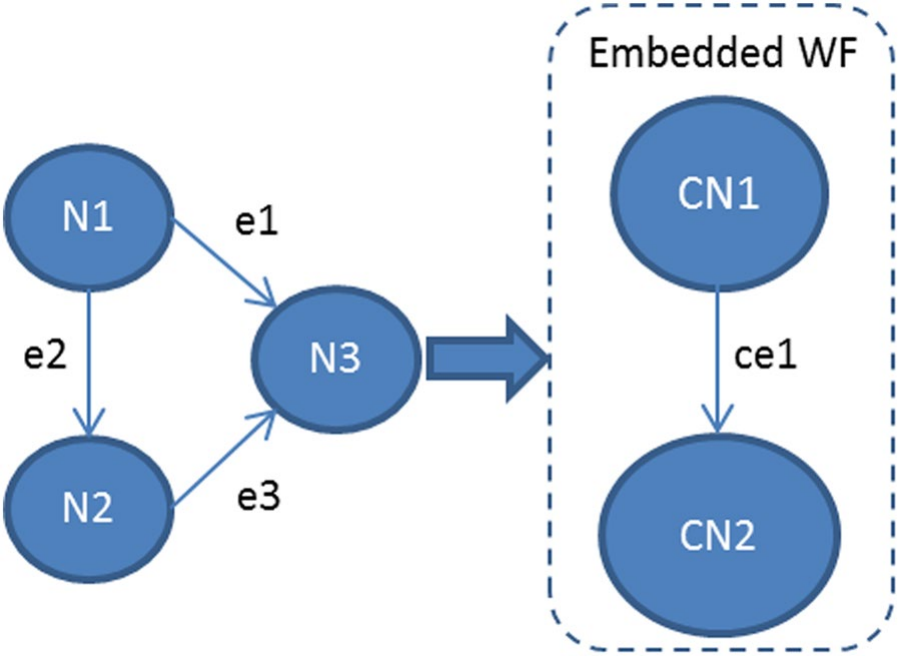
Example for a meta-workflow

To support workflow sharing we propose to incorporate atomic workflows as embedded workflows in meta-workflows to achieve more complex functionalities with less development efforts. In Fig. 1 the meta-workflow contains three jobs (N1, N2 and N3) and one embedded atomic workflow with node CN1 and CN2.

**Fig. 2:**
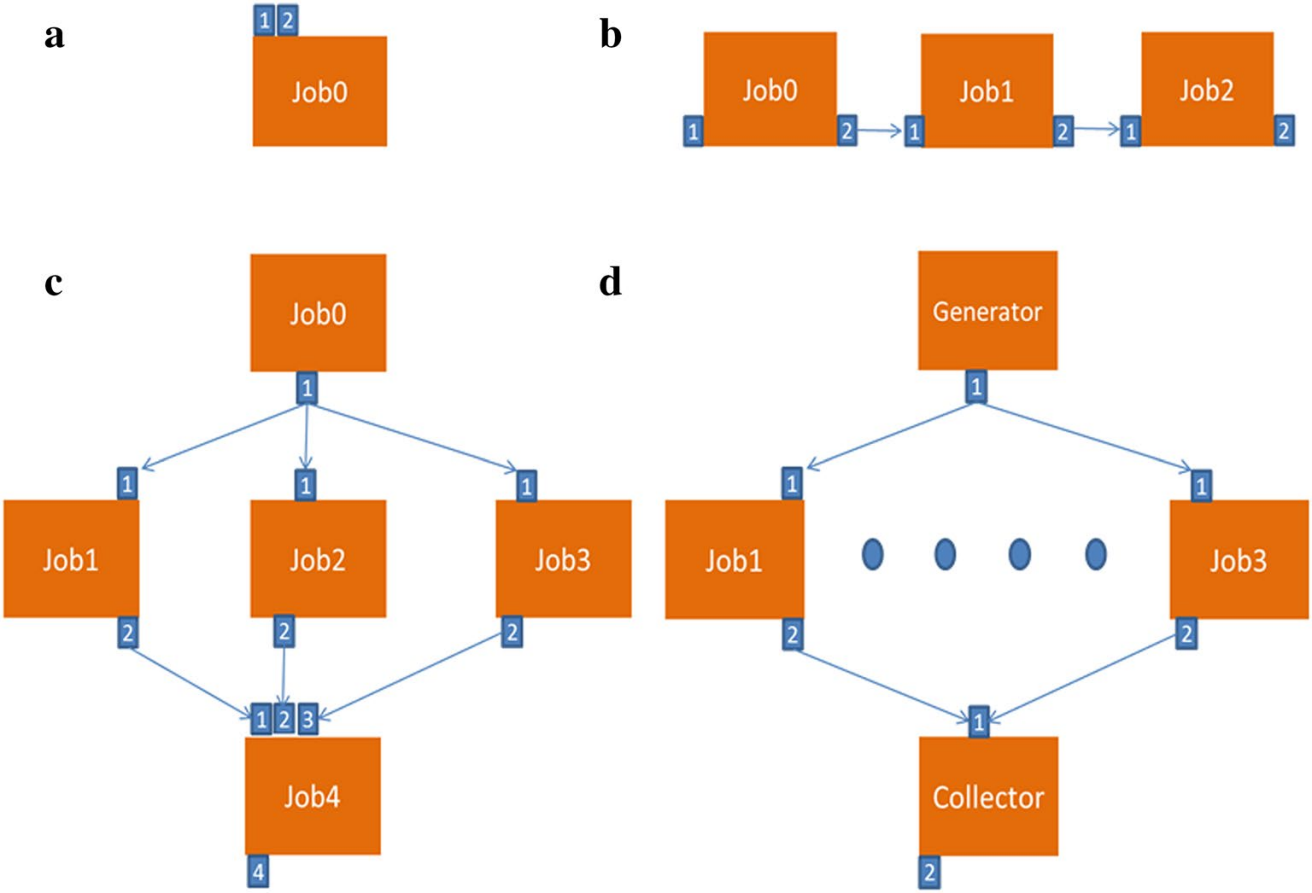
**a** Single job meta-workflow; **b** linear multi-job meta-workflow; **c** Parallel multi-job meta-workflow; **d** parameter sweep meta-workflow

As part of our efforts in [39], we identified different types of meta-workflows along with their formal definitions. These definitions are envisioned to make a significant contribution in supporting workflow developers to design new workflows by enabling them to comprehend the attributes and semantics of each type of meta-workflow. The different meta-workflow types are listed below with respective graphical representations from WS-PGRADE in Fig. 2a–d representing all workflow nodes as jobs that could be either workflow jobs or embedded workflows according to Eq. 7. Details of each of these can be found in [39].

1. *Single job meta*-*workflow (Fig.* 2 *a):* This is a special type of meta-workflow with a single job workflow. The job representing this workflow can be a simple job or an embedded workflow.
2. *Linear multi*-*job meta*-*workflow (Fig.* *2* *b):* This is a pipeline of multiple jobs in the native workflow system where any (or even all) of these jobs can be non-native workflows. The execution of each job depends on the receipt of inputs from previous jobs.
3. *Parallel multi*-*job meta*-*workflow (Fig.* 2 *c):* This is a workflow in the native workflow system that includes parallel branches. One or more of these branches can include one or more non-native workflows.
4. *Parameter sweep meta*-*workflow (Fig.* 2 *d):* The parameter sweep meta-workflow has a generator job which produces a number of inputs each to be consumed by a worker job. The collector job then aggregates the outputs of all the worker jobs and prepares the final output.

Considering that there could be multiple level of workflow embedding in meta-workflows we introduce *p* as the depth to describe it.

8 $$ {\text{M}}^{\text{p}}{-}{\text{WF}} = :\left\{ {{\text{J}}_{{{\text{WF}}1}} \ldots {\text{J}}_{\text{WFm}} ,\;{\text{WF}}_{{{\text{EM}}1}}^{{{\text{q}}1}} \ldots {\text{WF}}_{\text{EMn}}^{\text{qn}} ,} \right\} $$

where p—depth of the meta-workflow p = MAX{q_1_,…, q_n_}, q—depth of the embedded workflow q = 1, …, n

**Fig. 3:**
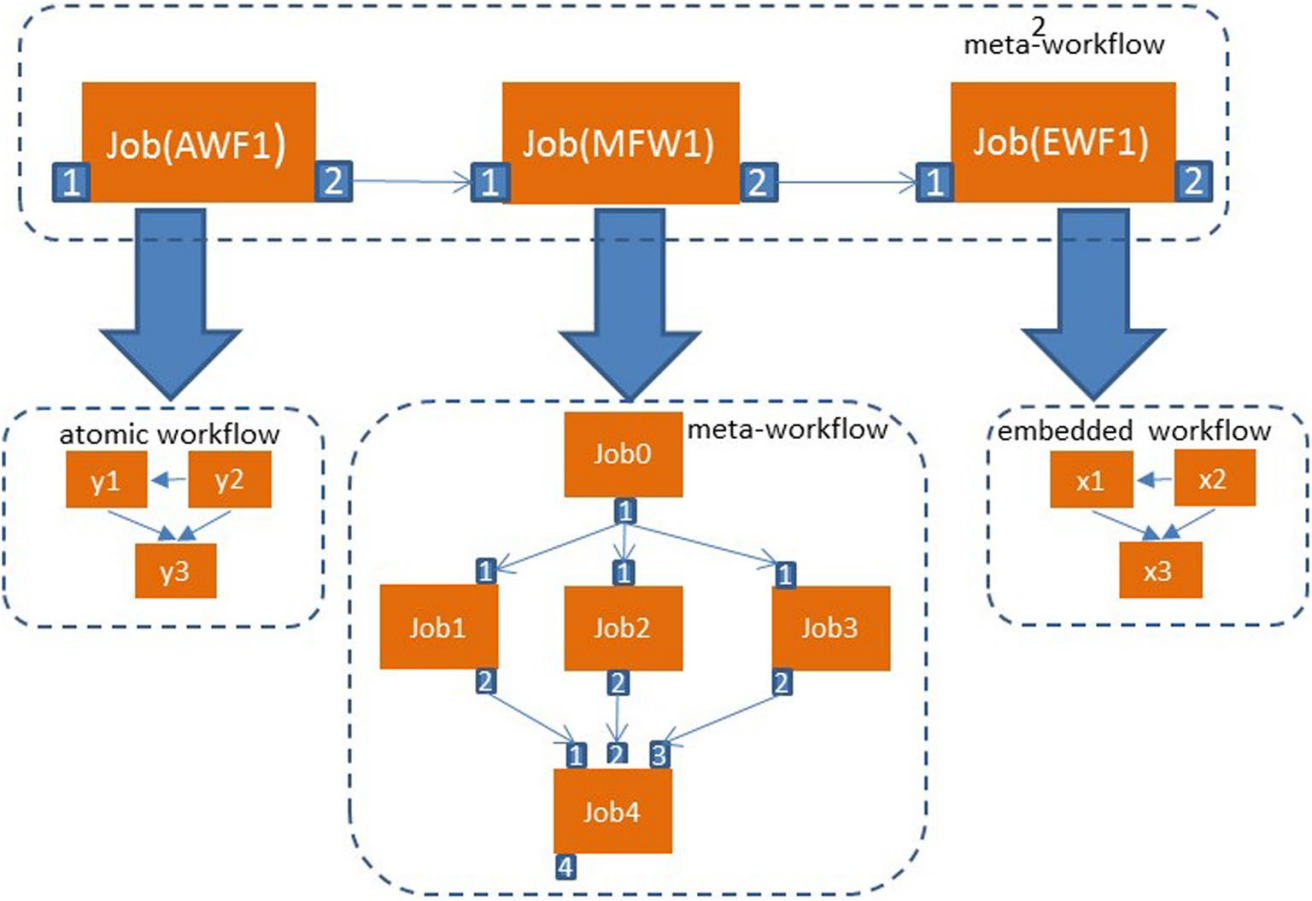
Meta-workflow of depth 2

Figure 3 presents a meta-workflow of depth 2. It combines an atomic workflow Job(AWF1), a meta-workflow Job(MWF1) and an embedded workflow Job(EWF1).

## Results and discussion

### Creating meta-workflows using atomic workflows

Two leading approaches for meta-workflow creation are the template-based approach to construct meta-workflows containing embedded workflows of the same workflow system and the black box based approach to develop meta-workflows incorporating embedded workflows of different workflow systems. Since we will use only WS-PGRADE workflows to outline how the Computational Chemistry community identifies and develops atomic workflows and constructs meta-workflows we will only describe the template based approach.

### Template based meta-workflow development

**Fig. 4:**
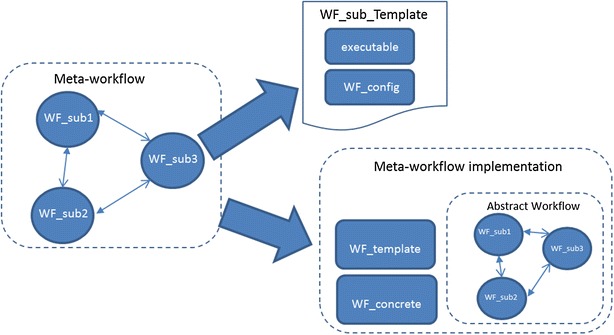
Template based approach for meta-workflow creation

**Fig. 5:**
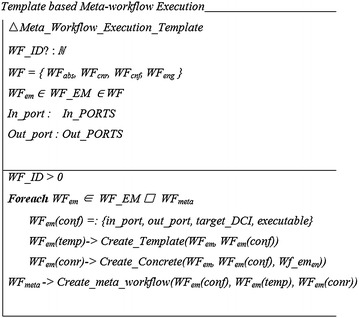
Template based meta-workflow creation

The template-based approach is focused at enabling re-use of existing workflows as embedded atomic workflows whilst allowing some degree of freedom for their customization. This approach introduces the concept of a *template,* which describes the default configuration of an embedded workflow. This configuration includes a number of parameters such as the input and output, data required for processing and the executables consequently serving as a prototype for use of the workflow. The template also controls the customization allowed for a workflow being shared. For instance, a workflow developer may allow customization of the data type of the input but restrict the number of input ports allowed for the workflow. This approach, therefore, offers more flexibility to a workflow developer in creating atomic workflows for sharing across different scientific disciplines without making the process cumbersome for the end user. Figures 4 and 5 presents a graphical representation of the template based approach for meta-workflow creation.

In order to formally describe the template based approach, we use the basic definition of the workflow given in (Eq. 1). In the context of the template based approach, we define a *template* to encompass the configuration of a workflow as

9 $$ {\text{WF}}_{\text{temp}} = :\left\{ {{\text{in}}\_{\text{port}},{\text{ out}}\_{\text{port}},{\text{ target}}\_{\text{DCI}},{\text{ executable}}} \right\} $$

Furthermore, as defined in Eq. 7 a meta-workflow is composed of multiple embedded workflows and multiple jobs. Therefore,

- WF_EM—set of embedded workflows for a meta-workflow, and
- WF_ID—unique ID for the workflow
- F_ID—unique ID for the workflow

The template-based approach has been implemented with the gUSE/WS-PGRADE science gateway technology as part of the ER-flow project [40]. In the current implementation, the first stage involves preparation of atomic workflows by the workflow developer. This includes defining the workflow graph, implementation of a concrete workflow with the defined workflow graph and building a template based on this implementation. This is followed by creation of an implementation for the concrete sub-workflow using the template. The creation of meta-workflow includes importing these sub-workflows shared via SHIWA Repository and configuring the required parameters such as the DCI where the workflow is intended to be processed, the executable and the data to be processed by the workflow.

### Quantum chemistry simulations

Quantum chemistry (QC) simulations deal with the electronic structure of molecules. An important task in quantum chemistry is the evaluation of the accuracy in describing specific molecular structures. Hence, lots of efforts are made in bench-marking studies with variation of functional and basis set in combination with solvent models and empirical dispersion correction. The job definition is always quite similar representing an ideal basis for the use of workflows. In a rather simple workflow, a given geometry can be calculated with a set of functionals and basis sets. The key geometric parameters are parsed and collected in tables afterwards, enabling direct comparison of the accuracy of the used methods. Another use case would be the study of a complex potential hypersurface by varying one or several geometric parameters. Then, a set of similar jobs has to be submitted to DCIs with the same functional and basis set but varying coordinates. Both types of workflows are independent of the quantum chemical code. Further post-processing can cover the addition of a solvent model, calculation of charges and frequencies, formatting of checkpoint files and definition of new job files for subsequent time-dependent DFT calculations. Quantum Chemistry workflows were primarily implemented in MoSGrid for Gaussian [41] and NWChem [42]. Both codes are used by novice and experienced users. Aiming at novice users, MoSGrid provides tutorials in the QC portlet on how to construct and submit a job and basic workflows are ready to use [18]. For experienced users, more complex workflows are available or can be assembled by themselves via the workflow portlet.

### Atomic operations versus atomic workflows in computational chemistry

Input is mostly the experimental structure obtained from single crystal X-ray diffraction analysis. The most fundamental step of every QC calculation is the geometry optimization where a converged wave function is calculated and the atomic positions are varied until all forces in the system come to a minimum. Afterwards, the frequency calculation checks whether a geometry represents a true minimum and delivers infrared frequencies of the compound. When dealing with systems containing metals, a very good accordance with the experimental structure is achieved when only 0.01 Å deviation in the chemical bond lengths is found. In many cases, the experimental optical properties of a given molecule must be compared to and explained by theoretical analysis. This is performed by time-dependent DFT calculations (TD-DFT) where the response of the wave function of the compound to an external periodic field (e.g. light) is simulated. More information about the electronic structure can be obtained by population analyses and charge calculations, e.g. by using natural bond orbitals (NBO) analysis [43]. These types of analyses allow to dissect the electron distribution and assign it to atoms in order to obtain partial charges, charge-transfer energies, hybridisation of atoms etc.

Previously, in QC, the calculation in gas phase was standard but today, to obtain a realistic description, solvent models are commonly applied. In explicit models, the single solvent molecules are modelled which leads to enormous computational effort as the number of particles in the simulation system increases exponentially. In implicit models, the solvents are simplified by their radius and their dielectric constant describing the continuum around the molecule of interest. The different approaches represent the compromise between best accuracy (explicit models) and highest speed (implicit models). Hence, every solvent has a specific set of parameters. Special attention has to be paid to the solvent description when changing the QC code or even only the version of the used code, as the implementations of solvent models vary. At the next level, dispersive interactions between molecules and parts of molecules must be described correctly. Dispersive interactions (London forces) are rather weak but they can change the relative energies between conformers since attractive forces between unipolar parts of molecules can affect the position of substituents. The dispersion model after Grimme adds pairwise interaction energies (DFT-D) to model possible contacts [44, 45]. It is highly important to understand the influences of different solvent and dispersion models on the structures, frequencies and energies of transition metal complexes because an accurateness of <0.1 kcal/mol is needed for a reasonable reaction mechanism prediction. Both enhancements can be added to all types of calculations described above.

Hence, candidates for atomic workflows are the following ones:

- geometry optimization
- frequency analysis
- time-dependent calculation
- population analysis
- charge calculation

### Quantum chemistry workflows

To evaluate how to use the atomic and meta-workflow concept in Quantum Chemistry, we identified several use cases. In this section, we present some of them in order to provide useful examples considering the current trends in Quantum Chemistry. Whenever similar job types of different molecules or different job types for the same molecule are to be submitted, a workflow can be an efficient and practical solution.

### Spectroscopic analysis

**Fig. 6:**
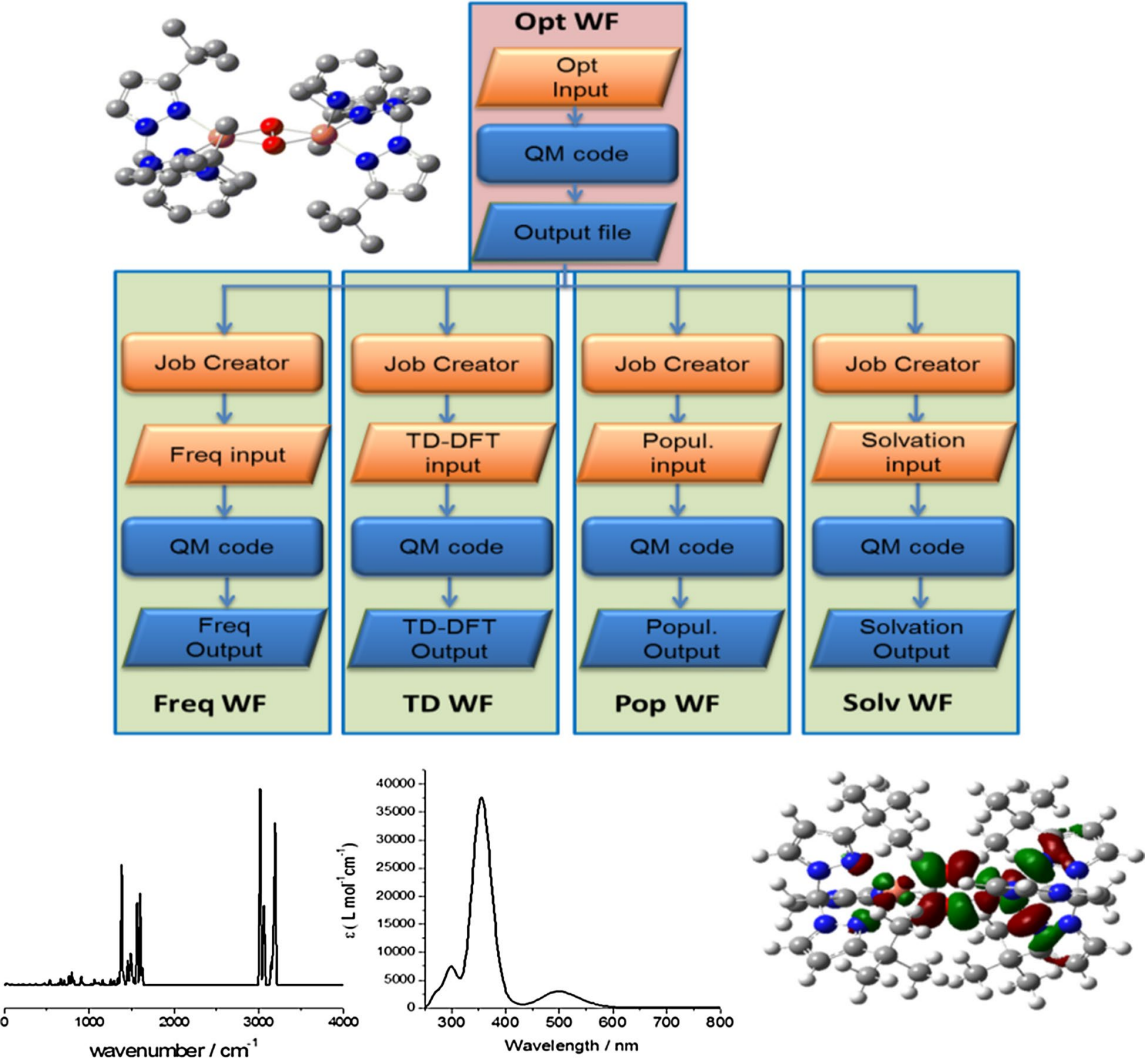
Spectroscopic analysis *Spec_Analy M*-*WF* meta-workflow (*top left* molecular structure, bottom from *left* to *right*: Raman spectrum, UV/Vis spectrum, lowest unoccupied orbital)

In this context, a highly interesting use case is the so-called spectroscopic analysis (Fig. 6). After a first geometry optimization of the selected molecule several further simulations such as frequency analysis, time dependent calculation, population analysis and solvent analysis are performed using the optimized coordinates. These simulations can be further divided into smaller tasks, such as the input file generation by a so-called *job generator*, then the job submission to the DCI and the calculation by the QC code, which produces the corresponding output. Input and output files are graphically represented by rhombs whereas jobs are rounded boxes.

To create a spectroscopic analysis meta-workflow (*Spec_Analy M*-*WF*) first, we defined the structure of the meta-workflow. Next, we identified the atomic workflows i.e. small operations or tasks that can be re-used in other meta-workflows. The first atomic workflow (*Opt WF*) runs a simple geometry optimization. The subsequent atomic workflows have a similar structure but they provide different functionalities. They contain a converter script that extracts the output geometry from the optimization output and combines it with blank input files (i.e. just lacking input coordinates) with the corresponding keywords for frequency calculations (*Freq WF*), time-dependent DFT calculations giving UV/Vis spectra (*TD WF*), population analyses (*Pop WF*) and subsequent calculations in solvents (*Solv WF*). All these atomic workflows, shown in Fig. 6 are highly valuable since they can be re-used in other QC meta-workflows.

With regard to a real-life system as depicted in Fig. 6, the spectroscopic analysis has to tackle issues such as antiferromagnetic coupling between copper atoms, correct description of the coordination sphere and multiple conformations of the whole molecule. [46, 47] Methodologically, density functional theory is most appropriate here due to size of the system and investigated questions.

**Fig. 7:**
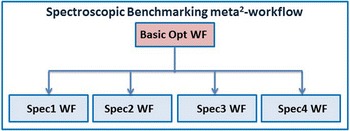
*Spec_Bench M* ^*2*^-*WF* spectroscopic benchmarking meta-workflow

Hence, the spectroscopic analysis meta-workflow needs to be performed several times for an array of functionals and basis sets which have to be tested for the ultimate structural and optical description with respect to experimental data. Now, this meta-workflow can be combined into a spectroscopic benchmarking meta^2^-workflow (*Spec*-*Bench M* ^*2*^-*WF*). Figure 7 shows four spectroscopic analysis meta-workflows which are combined after performing a basic optimization. This basic optimization atomic workflow (*Basic Opt WF*) runs a pre-optimization step, which saves calculation time in all subsequent optimizations included in the spectroscopic workflows (Spec1 WF…Spec4WF). The *Basic Opt WF* can for example use a smaller basis set. Here, the *Opt WF* from Fig. 6 can be re-used. The *Spec*-*Bench M* ^*2*^-*WF* saves a lot of time in this application.

After execution of the spectroscopic benchmarking meta-workflow, the user collects and analyses the data. These steps should still be performed manually since the outputs are rather diverse. But the largest benefit of using meta-workflows is that it decreases the overall workload of defining all input job files, extracting geometry data after the pre-optimization step and others, as a result it saves a lot of the researchers´ time.

### Optical benchmarking

**Fig. 8:**
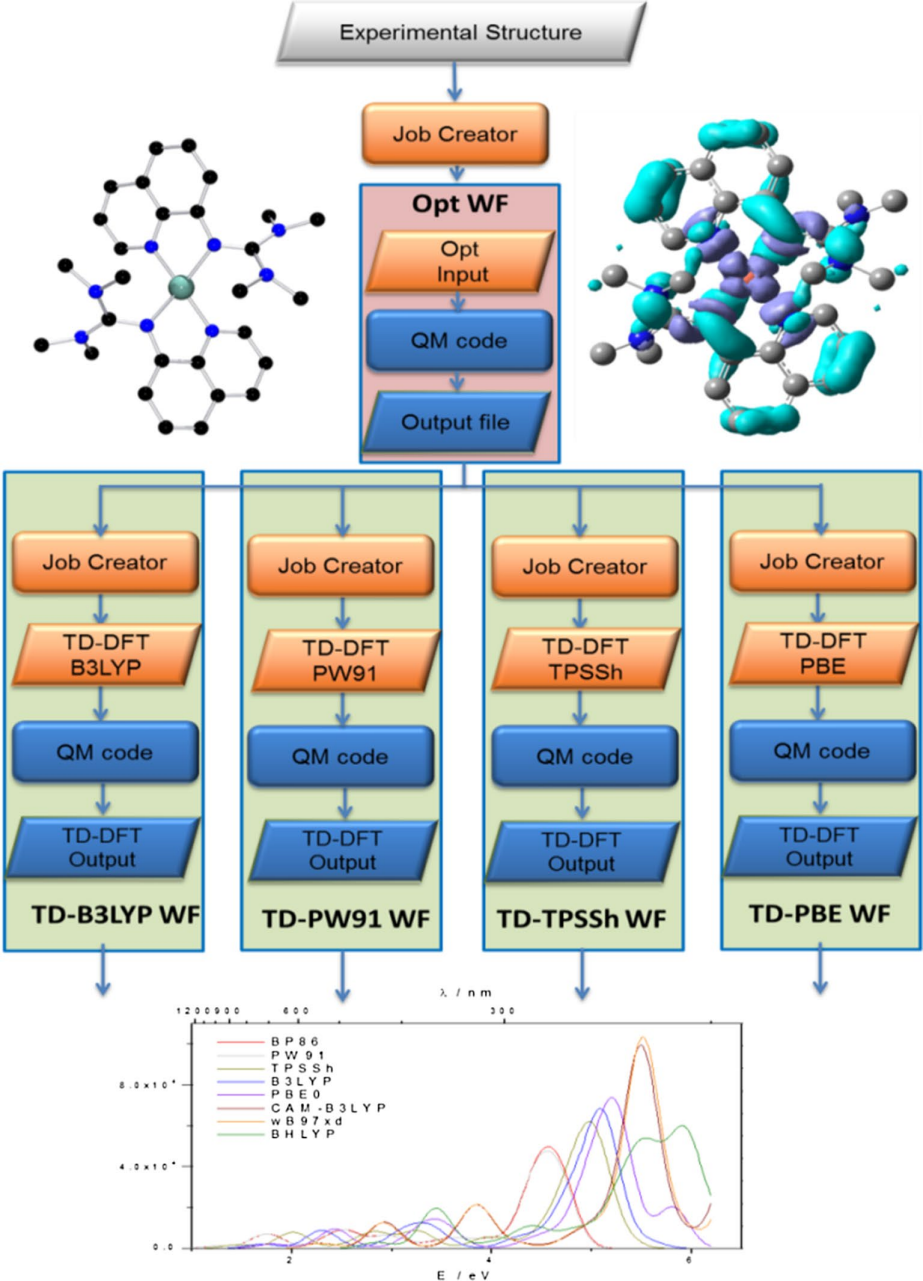
*Opt_Bench M*-*WF* optical benchmarking meta-workflow with five atomic workflows yielding theoretical UV/Vis spectra

The TD-DFT calculations can be highly dependent on the selection of the functional. Hence, an optical benchmarking may be needed to investigate the influence of the functional on the prediction of the optical transitions. Charge-transfer transitions are very sensitive towards the choice of the functional [48–52] and the results can largely deviate from the experimental spectrum. Hence, for a new transition metal system, one always needs to perform a so-called optical benchmarking and find a suited functional to describe measured spectra correctly. After optimizing the structure, different functionals, such as GGAs, meta-GGAs, hybrid-GGAs can be used to evaluate the functional dependency of the optical transitions (Fig. 8). In detail, this might be B3LYP, PW91, TPSSh, PBE, to name just a few.

Each TD calculation can be implemented as a separate atomic workflow. See atomic workflows in Fig. 8: *TD*-*B3LYP WF, TD*-*PW91 WF, TD*-*TPSSh*-*WF, TD*-*PBE WF*. They run different time-dependent DFTs (TD-DFT). Having these atomic workflows and the *Opt WF* we have created the *Opt*-*Bench M*-*WF* meta-workflow. The strength of this concept lies in the re-usability of the TD atomic workflows which have been tested successfully and collected in the MoSGrid Repository. Moreover, for every step, metadata is annotated and stored facilitating the organization of the computational chemists work. In principle, this optical benchmarking meta-workflow can be conceptualized in a broader way when more functionals are required to describe a complicated electronic behaviour [48–50].

### Structural benchmarking

**Fig. 9:**
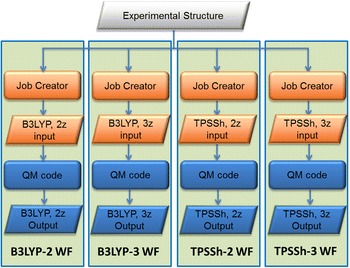
*Geo_Opt M*-*WF* small structural benchmarking meta-workflow

Functionals also influence the structural details of molecules. Hence, a benchmarking for the structural influence (Fig. 9) can include variation of the functional and of the basis set. To perform this benchmarking we can create a meta-workflow (*Geo_Opt M*-*WF*) with different types of optimization runs. The basis set is indicated by “2z” and “3z” which denotes the quality of the basis set. Larger basis sets give better agreement with experimental structural information but the calculation time can increase to such an extent that the calculation might no longer be feasible when dealing with molecules of more than 200 atoms. To run structural benchmarking a combination of a particular function and the quality of the basis set can be implemented as atomic workflows, for example: *B3LYP*-*2 WF, B3LYP*-*3 WF, TPSSh*-*2 WF, TPSSh*-*3 WF*, etc. (See in Fig. 9).

**Fig. 10:**
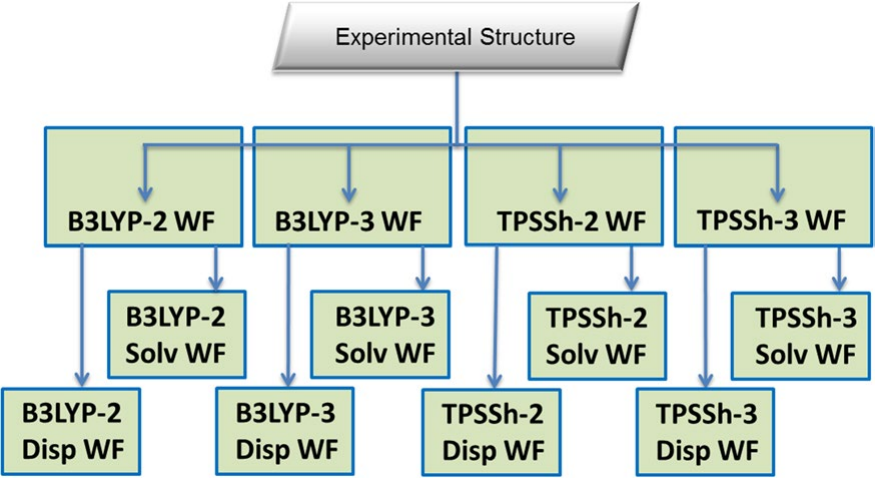
*Struc_Bench M* ^*2*^-*WF* structural benchmarking meta^2^-workflow using dispersion and solvent models

Further, this meta-workflow can easily be extended with post-optimization steps using dispersion correction or solvent models (Fig. 10) in a meta^2^-workflow (*Struc_Bench M* ^*2*^-*WF*). These steps can be implemented by B3LYP and TPSSh atomic workflows that run the dispersion corrections and solvent models.

**Fig. 11:**
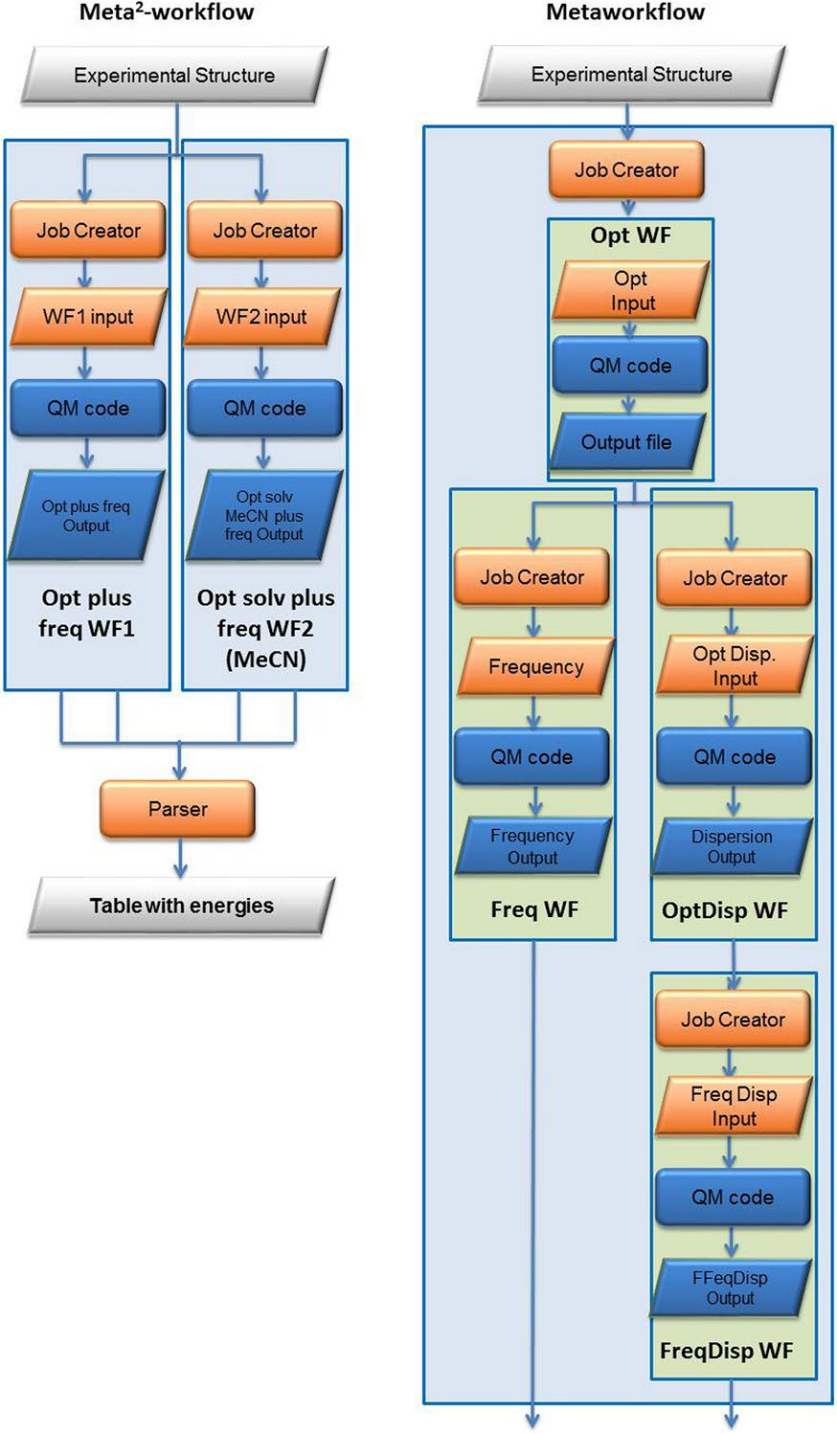
Left: *Struc_Opt M* ^*2*^-*WF* meta^2^ -workflow for frequency optimization in dispersion and solvent, right: *Freq_Disp_Opt M*-*WF* meta-workflow for frequency optimization in dispersion

Taking into account, that the chemist finally needs the frequencies of desired molecules, frequency and optimization tasks should be combined together with the options of dispersion and solvent models. Right in Fig. 11 there is a three-layer meta-workflow (*Freq_Disp_Opt M*- *WF*) that incorporates four atomic workflows. First, the geometry of the selected molecule is optimized by the *Opt WF* atomic workflow. Next, two atomic workflows are executed in parallel calculating the vibrational properties of the molecule by the *Freq WF* atomic workflow and the optimized molecule is re-optimized using dispersion by the *OptDisp WF* atomic workflow. Finally, the corresponding frequencies of the re-optimized molecule are calculated by the *FreqDisp WF* atomic workflow.

The *Freq_Disp_Opt M*- *WF* meta-workflow runs a simulation in gas phase. It can be combined with the *Freq_Solv Opt M*-*WF* meta-workflow to run a simulation in a common solvent as polarizable continuum model into a meta^2^-workflow. The *Struc_Opt M* ^*2*^-*WF* meta workflow saves a lot of time of manual job definition and result analysis, easily up to a factor of 10 in terms of the researchers working time.

### Inorganic polymerization catalysis

**Fig. 12:**
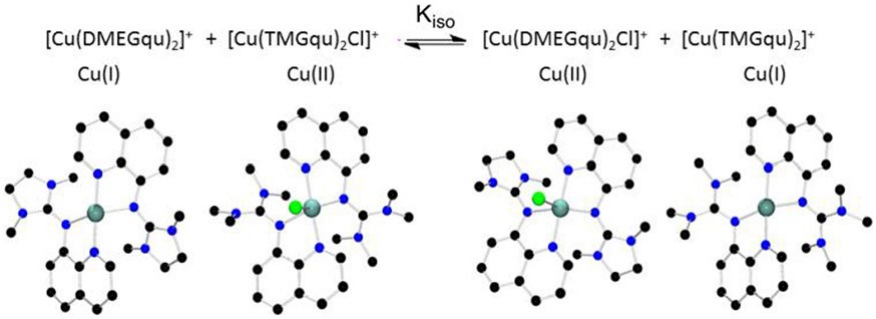
Equilibrium between copper(I) and (II) complexes with guanidine–quinoline ligands and the structures of these complexes

Since QC is applied in all fields of chemistry, we searched for a use case from catalysis in order to demonstrate the wide applicability of the concept. In modern controlled polymerization techniques such as atom transfer radical polymerization (ATRP) [53], the control over redox properties is crucial for the polymerization control. In this use case, we are interested in the relative ratio of an equilibrium between different copper guanidine complexes which can interchange electrons and halide atoms. Hence, we have two slightly differing ligands, TMGqu and DMEGqu [54], which stabilise copper(I) and copper(II) complexes. As equilibrium with the equilibrium constant K_iso_, we can write the atom transfer reaction as described in Fig. 12. We start with experimental structures of the four complexes. So, the whole equilibrium workflow is performed for each structure.

**Fig. 13:**
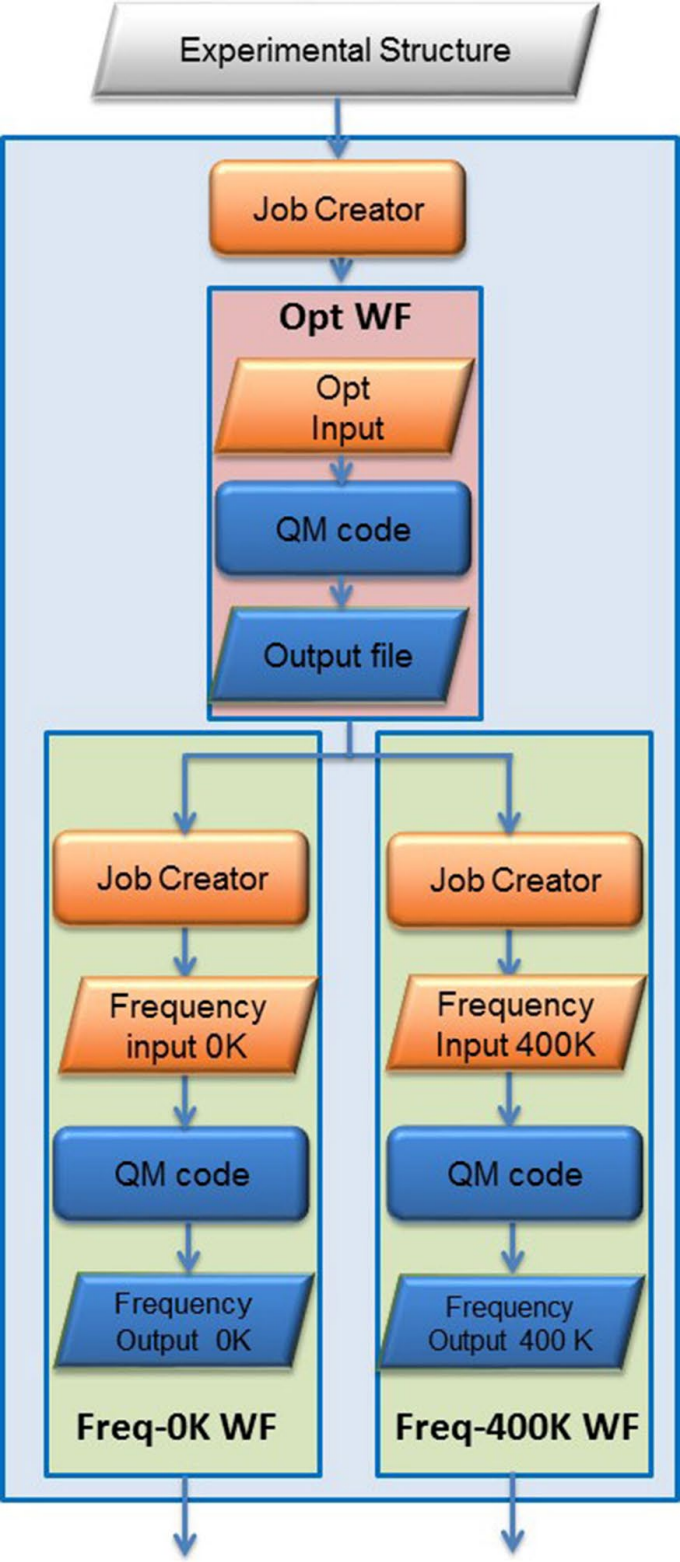
*Equil_Calc M*-*WF* meta-workflow with three atomic workflows

To describe the equilibrium we developed the *Equil_Calc WF* meta-workflow with a three-layer structure as depicted in Fig. 13. There are three atomic workflows to run the QC code at the bottom layer (or first layer). The *Opt WF* atomic workflow processes the *input file* (of the experimental structure) produced by the *Job Creator* from the experimental structure and generates an optimization input file within this atomic workflow. The optimized structure is parsed by the job creator of the *Freq*-*0* *K WF* and *Freq*-*400* *K WF* atomic workflow to create two parallel frequency files (Fig. 13). Normally, the standard frequency calculations are performed at 0 K but in this equilibrium case, experimental conditions at 400 K shall be considered as well. These two atomic workflows run in parallel. This meta-workflow represents the second layer of the simulation.

**Fig. 14:**
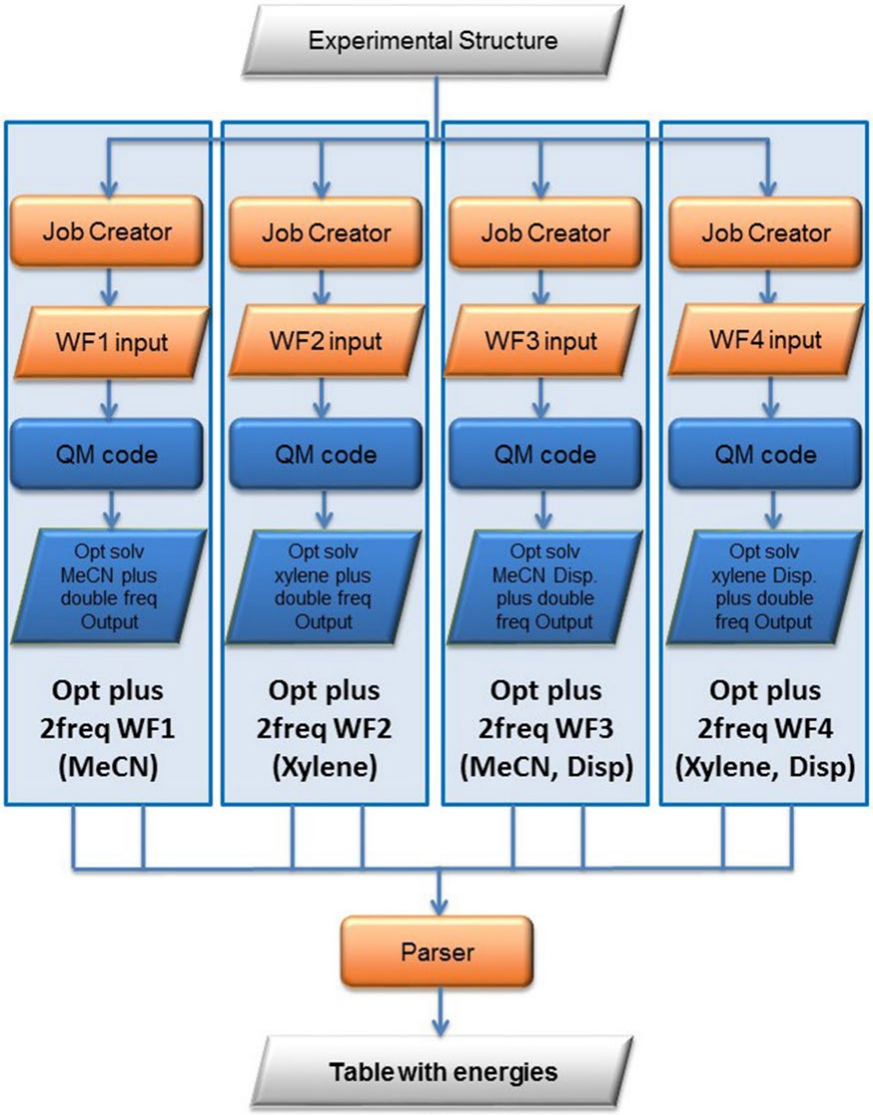
*Equil_Solv M* ^*2*^-*WF* meta^2^workflow

As this type of calculations has to be performed in different solvents (acetonitrile = MeCN and xylene) as well as with and without dispersion, the meta-workflow has to be performed four times in parallel inside the *Equil_Solv_M* ^*2*^-*WF* meta workflow given in Fig. 14. This meta-workflow represents the third layer of the simulation incorporating the *Opt plus 2freq M*-*WF* meta-workflows. The result of this meta-workflow is a table containing the energies, enthalpies and free energies parsed out of the result files of the eight frequency jobs, since every single meta-workflow produces two frequency output files. This table contains basically the results for one complex.

**Fig. 15:**
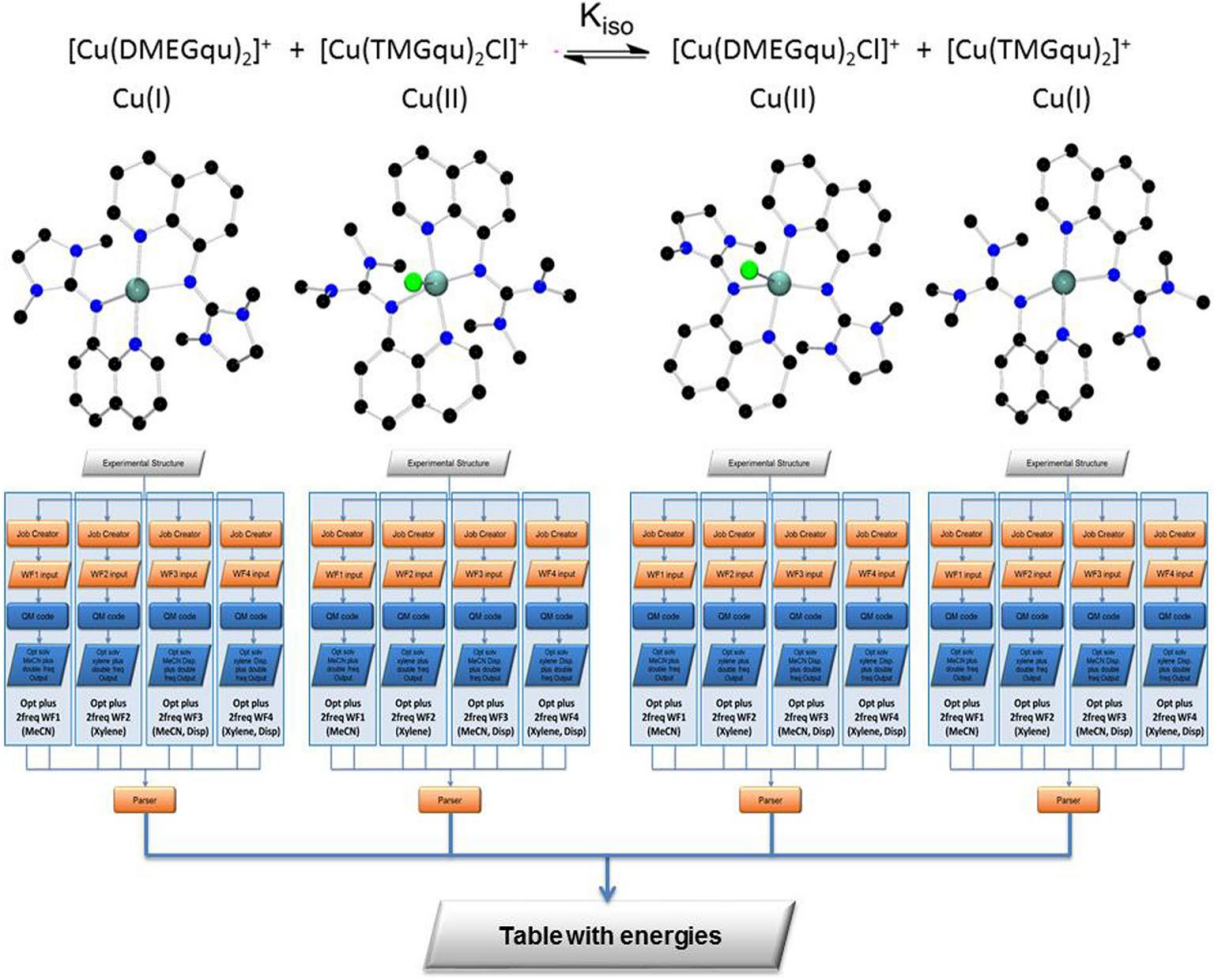
*Equil_Energ M* ^*3*^-*WF* meta^3^workflow defined for each complex and analysed into a single table

After completing the *Equil_Solv M* ^*2*^-*WF* meta^2^-workflow the user can summarize the resulting tables and calculate the relative energies yielding the desired K_iso_ value. This could be included into *the Equi_Energ M* ^*3*^-*WF* meta-workflow at the fourth workflow layer (Fig. 15). In principle, one should also evaluate the functional influence because different density functionals treat electron correlation differently yielding different results here. This would even add a fifth layer.

In the daily chemical computational work, we have found that every layer adds efficiency with a factor of around 2–3 as time-consuming job definition, structure extraction and data collection are considerably facilitated. This factor can be calculated by the following example: the manual definition of one job takes 3 min. So, the embedded workflow in Fig. 13 would need 9 min and the meta-workflow in Fig. 14 four times more, 36 min. The highest level WF would then add up to 144 min of job definition time in manual mode. The data extraction time can be assumed to the same amount; hence, we end up with approximately 280 min for one run of the jobs summarized in Fig. 15. In fact, the corresponding workflow needs to be defined and tested, but since the embedded workflows are very similar the meta-workflow system saves time, such that the whole workflow definition needs 3–4 h. But then, it can be performed several times and re-used in itself or by its building blocks. Thereby, we estimate the efficiency factor to 2–3.

### Workflow libraries in quantum chemistry simulations

In the ER-flow project [40] the Computational Chemistry community developed 26 atomic workflows and 12 meta-workflows, presented in Additional file 1: Table S1–S6, to run optical and structural benchmarking, spectroscopic simulations and investigations on inorganic polymerization catalysts experiments. Considering these workflows we created the Quantum Chemistry workflow library with five sub-libraries (Table 2).

**Table 2:** Structure of the quantum chemistry workflow library

Workflow library	Sub-libraries
DOMAIN—quantum chemistry	SUB-DOMAIN_1_—basic operations
	SUB-DOMAIN_2_—spectroscopic analysis
	SUB-DOMAIN_3_—optical benchmarking
	SUB-DOMAIN_4_—structural benchmarking
	SUB-DOMAIN_5_—inorganic polymerization catalysis

Table 3 presents the basic operations sub-library. It contains the atomic workflows that implement basic Quantum Chemistry operations and can be used in scientific experiments of different sub-domains in QC. These atomic workflows are highlighted in bold in Additional file 1: Table S1–S6. For example the *Opt WF* atomic workflows is incorporated in the *Spect_Analy M*-*WF, Opt_Bench M*-*WF, Freq_Opt M_WF* and *Equil_Calc M*-*WF* meta-workflow, while the *Freq WF* atomic workflow in the *Spec_Analy*_ and *Freq_Opt M*-*WF* meta-workflow.

**Table 3:** Atomic workflows of the basic operations sub-library

Atomic workflows	Functionality
Opt WF	Geometry optimization
Basic Opt WF	Geometry optimization with small basis set
Freq WF	Frequency calculation
TD WF	Time-dependent DFT calculation
Pop WF	Population analysis
Solv WF	Optimization in solvent

The other four sub-libraries contain atomic workflows that deliver operations specific to a particular Quantum Chemistry sub-domain, for example optical benchmarking, structural benchmarking, etc. These atomic workflows are listed in Additional file 1: Table S1–S6.

## Conclusions and further works

Herein, we have shown that re-occurring tasks in Quantum Chemistry scientific experiments can be facilitated by re-using and sharing workflows. We introduce and formally describe the concept of the atomic workflow. Atomic workflows represent basic operations for example optimization, frequency calculation, population analysis, etc. in Quantum Chemistry. Since these operations are performed in multiple scientific experiments they can be shared among these experiments. We also propose to build workflow libraries that manage and publish atomic workflows. Workflow libraries are domain specific, i.e. each scientific domain may have its own workflow library with several sub-domains. Having atomic workflows researchers can combine them into complex workflows called meta-workflows. We propose to use atomic workflows as building blocks for complex meta^n^-workflows. We extend the existing formal description of meta-workflows to support sharing atomic workflows incorporating them into meta-workflows. The Quantum Chemistry uses among others the WS-PGRADE workflow systems to create workflows and run scientific workflows through science gateways such as the MoSGrid Portal and the SHIWA Portal. We developed and formalized a template based approach to create WS-PGRADE meta-workflows to incorporate atomic workflows. Chemists, who become acquainted to workflows, can apply this technology to scientific problems. The work of dissecting a chemical theoretical problem into basic operations and defining the relevant atomic workflows is illustrated by spectroscopic analysis, optical and structural benchmarking and inorganic polymerization catalysis analysis workflows. We have created and uploaded 26 atomic workflows into the Quantum Chemistry workflow library that contains five sub-domains. We incorporated these atomic workflows into 12 meta-workflows. Considering developing atomic workflows and incorporating them into meta-workflows we can conclude that the re-use of the atomic workflows significantly decreases the efforts and time needed for creating scientific experiments. As a result, it makes the research more efficient. In future work, we plan to apply this concept to more complex scientific experiments where input preparation and output parsing is more involved

## Supporting Information

The Supporting Information contains details on the atomic and meta-workflows.

## Additional file

**Additional file 1.** The supporting information contains details on the atomic and meta-workflows.
