# Supplementary material for: Multi-level meta-workflows: new concept for regularly occurring tasks in quantum chemistry
Source: J Cheminform. 2016 Oct 20;8:58. doi: 10.1186/s13321-016-0169-8 (PMC5073744; doi:10.1186/s13321-016-0169-8)
Supplement: Supplementary file 1 — Additional file 1. The supporting information contains details on the atomic and meta-workflows. [file 13321_2016_169_MOESM1_ESM.pdf]

Multi-level Meta-workflows: New Concept for Regularly Occurring Tasks in Quantum Chemistry

Junaid Arshad,[a] Alexander Hoffmann,[b] Sandra Gesing,[c] Richard Grunzke,[d] Jens Krüger,[e] Tamas Kiss,[a] Sonja Herres-Pawlis,[b]* Gabor Terstyanszky [a]*

[a] Centre for Parallel Computing, School of Electronics and Computer Science University of Westminster, 115 New Cavendish Street, London W1W 6UW, UK. terstyg@westminster.ac.uk
[b] Institut für Anorganische Chemie, Lehrstuhl für Bioanorganische Chemie, RWTH Aachen University, Landoltweg 1, 52074 Aachen, Germany, sonja.herres-pawlis@ac.rwth-aachen.de
[c] University of Notre Dame, 123 Information Technology Center, Notre Dame, IN 46556, USA
[d] Center for Information Services and High Performance Computing, Technische Universität Dresden, Zellescher Weg 12-14, 01062 Dresden, Germany
[e] Applied Bioinformatics Tübingen, University of Tübingen, Sand 14, 72076 Tübingen, Germany

name	functionality
atomic workflows	
Opt WF	geometry optimization
Basic Opt WF	geometry optimization with small basis set
Freq WF	frequency calculation
TD WF	time-dependent DFT calculation
Pop WF	population analysis
Solv WF	optimization in solvent
meta-workflows	
Spec M-WF	spectroscopic analysis
Spec-Bench M^2-WF	spectroscopic benchmarking

Table S1: Spectroscopic simulation workflows (Fig. 6 and 7)

name	functionality
**atomic workflows**	
**Opt WF**	geometry optimization
TD-B3LYP WF	TD calculation with B3LYP
TD-PW91 WF	TD calculation with PW91
TD-TPSSh WF	TD calculation with TPSSh
TD-PBE WF	TD calculation with PBE
**meta-workflows**	
Opt_Bench M-WF	optical benchmarking

Table S2: Optical benchmarking workflows (Fig. 8)

name	Functionality
atomic workflows	
B3LYP-2 WF	geometry optimization with B3LYP and 2z basis set
B3LYP-3 WF	geometry optimization with B3LYP and 3z basis set
TPSSh-2 WF	geometry optimization with TPSSh and 2z basis set
TPSSh-3 WF	geometry optimization with TPSSh and 3z basis set
meta-workflows	
Geo_Opt M-WF	geometry optimization benchmarking

Table S3: Structural benchmarking workflows (Fig. 9)

name	functionality
atomic workflows	
B3LYP-2 Solv WF	geometry optimization with B3LYP and 2z basis set and solvent model
B3LYP-2 Disp WF	geometry optimization with B3LYP and 2z basis set and dispersion
B3LYP-3 Solv WF	geometry optimization with B3LYP and 3z basis set and solvent model
B3LYP-3 Disp WF	geometry optimization with B3LYP and 3z basis set and dispersion
TPSSh-2 Solv WF	geometry optimization with TPSSh and 2z basis set and solvent model
TPSSh-2 Disp WF	geometry optimization with TPSSh and 2z basis set and dispersion
TPSSh-3 Solv WF	geometry optimization with TPSSh and 3z basis set and solvent model
TPSSh-3z Disp WF	geometry optimization with TPSSh and 3z basis set and dispersion
meta-workflows	
Struct_Bench M^2-WF	structural benchmarking

Table S4: Structural benchmarking workflows (Fig. 10)

name	functionality
atomic workflows	
**Opt WF**	geometry optimization
**Freq WF**	frequency calculation
OptDisp WF	dispersion optimization
FreqDisp WF	frequency-dispersion calculation
meta-workflows	
Freq_Disp_Opt M-WF	gas phase optimization
Freq_Solv_Opt M-WF	solvent phase optimization
Struc_Opt M^2-WF	structural optimization

Table S5: Structural benchmarking workflows (Fig. 11)

name	functionality
atomic workflows	
**Opt WF**	geometry optimization
Freq-0K WF	frequency calculation at 0 K
Freq-400K WF	frequency calculation at 400 K
meta-workflows	
Equil_Calc M-WF	Structure optimization for equilibrium calculation
Opt plus 2 Freq M-WF	temperature-dependent energies calculation
Equil_Solv M^2-WF	Structure optimization in different solvents
Equil_Energ M^3-WF	Combination for all complexes in equilibrium

Table S6: Inorganic polymerization catalysis workflows (Fig. 14 and 15)
